# Multiple ocular manifestations in a patient diagnosed with herpes zoster ophthalmicus: case report

**DOI:** 10.22336/rjo.2024.16

**Published:** 2024

**Authors:** David-Ionuț Beuran, Mioara-Laura Macovei, Ioana Ruxandra Boca

**Affiliations:** * Department of Ophthalmology, „Dr. Carol Davila” Central Military University Emergency Hospital, Bucharest, Romania; ** „Carol Davila” University of Medicine and Pharmacy, Bucharest, Romania; *** Clinical Emergency Eye Hospital, Bucharest, Romania

**Keywords:** herpes zoster ophthalmicus, episcleritis, uveitis, antivirals

## Abstract

**Objective:** Our purpose was to present a case of a patient diagnosed with herpes zoster ophthalmicus with multiple ocular manifestations.

**Case presentation:** A 70-year-old Caucasian male presented to the hospital for headache and skin hyperesthesia on the scalp and forehead on the left side. The diagnoses of herpes zoster ophthalmicus and acute conjunctivitis were made for the left eye. The patient was followed up for 6 months and during that period the following diagnoses were made for the same eye: peripheral sterile corneal infiltrates, episcleritis, and hypertensive anterior uveitis.

**Discussions:** Herpes zoster ophthalmicus occurs when the reactivation of the dormant virus involves the ophthalmic division of the trigeminal nerve. The most frequent ocular presentations are conjunctivitis, keratitis, uveitis, episcleritis, and scleritis. The standard therapy consists of antivirals, such as acyclovir, valacyclovir, and famciclovir to limit the replication of the virus. The patient’s risk factors, the course of treatment, and the severity of the disease, all affect the prognosis, which is highly variable. Prevention of the disease consists of vaccination with one of the following two vaccines, Zostavax and Shingrix.

**Conclusions:** Final visual acuity for the left eye remained 1 despite numerous manifestations of the disease.

**Abbreviations:** VZV = Varicella-zoster virus, BCVA = best-corrected visual acuity, OU = both eyes, OD = right eye, OS = left eye, IOP = intraocular pressure, NCT = non-contact tonometer, ZVX = Zostavax vaccine

## Introduction

*Virus structure*

Varicella-zoster virus (VZV) is a part of the Herpesviridae family and represents the etiologic agent of varicella (primary disease) and herpes zoster (secondary disease). The virus has an envelope, a double-stranded deoxyribonucleic acid, and establishes latency. Some precise glycoproteins mediate the cell and humoral immunity to VZV [**1**]. 

*Transmission*

VZV is very contagious and the virus is transmitted via respiratory tract or from direct contact with vesicular lesions, causing the primary infection, which is varicella. The incubation time ranges between 10-21 days [**2**,**3**].

*Epidemiology*

VZV occurs worldwide and is less frequent in some developed countries due to the vaccine. The incidence of varicella depends on the type of climate, for example in temperate climates the peak of the disease occurs before adolescence. There is also a higher incidence during winter and spring [**4**]. On the other side, herpes zoster also occurs worldwide, but the incidence increases with age and with the presence of risk factors such as immunosuppression, infections, and mental stress [**2**].

*Primary infection*

The primary disease manifests with cutaneous lesions that are in different developing stages. These are infectious until the vesicular lesions become dry, but the host is contagious up to 24-48 hours before the initial skin eruption [**1**,**3**].

*Secondary infection*

After the initial infection, the virus remains latent within the sensory ganglia for a variable amount of time. It can be reactivated, due to many factors (e.g. increased age, stress, immunosuppression) causing extraocular and ocular manifestations of herpes zoster [**1**,**5**].

## Case presentation

A 70-year-old Caucasian male presented to the hospital for headache and skin hyperesthesia on the scalp and forehead on the left side. The symptoms started a week before the presentation. From personal history, we should mention chicken pox. Clinical examination revealed eruption on the scalp, forehead, and periocular (dermatome V1), on the left side, respecting the midline, currently represented by well-defined brown macules. Five days before, the lesions were represented by blisters.

Best-corrected visual acuity (BCVA) was 1 in both eyes (Oculus Uterque: OU) after correction of compound hyperopic astigmatism with the following refraction, right eye (Oculus Dexter: OD) +1,75/-1,00/2° and left eye (Oculus sinister: OS) +1,50/-0,75/59°. The intraocular pressure (IOP) in the right eye was 18 mmHg non-contact tonometer (NCT) and in the left eye 16 mmHg NCT. At anterior pole examination, both eyes presented pseudoexfoliative material at the level of the anterior lens capsule and cortical lens opacities. OS revealed eyelid edema, conjunctival hyperemia, and watery secretions. At posterior pole examination, OD presented small, white vitreous opacities and OS was without pathological changes.

After the initial examinations, the following diagnoses were established for OS: Herpes zoster ophthalmicus, acute conjunctivitis; for OD: asteroid hyalosis and for OU: age-related cataract, pseudoexfoliation syndrome, compound hyperopic astigmatism, presbyopia.

Blood was prelevated for the following: hemogram, fibrinogen, erythrocyte sedimentation rate, lipid profile, serum urea, uric acid, creatinine, blood glucose, alanine transaminase, and aspartate aminotransferase. Those outside normal limits were: fibrinogen 460 mg/dL, erythrocyte sedimentation rate 23 mm/1h, and blood glucose 185 mg/dL. Dermatology consultation confirmed the diagnosis of herpes zoster ophthalmicus and treatment was initiated for the skin lesions. Furthermore, the diabetology consultation confirmed the diagnosis of type II diabetes.

Considerations in the differential diagnosis of the rash included the eruption from the herpes simplex virus, which usually occurs in younger patients. The vesicles are grouped in a bouquet, situated on an erythematous background, with a limited distribution that does not respect the dermatomes. 

A systemic treatment was initiated with oral acyclovir tablets, 800 mg five times daily for ten days. The local treatment for the skin lesions included fusidic acid ointment, 20 mg/g, applied externally on lesions twice a day for ten days. Topical treatment for OS contained ganciclovir ophthalmic gel 1,5 mg/g three applications daily for ten days and preservative-free artificial tears four times daily for ten days and later as needed. 

**Fig. 1:**
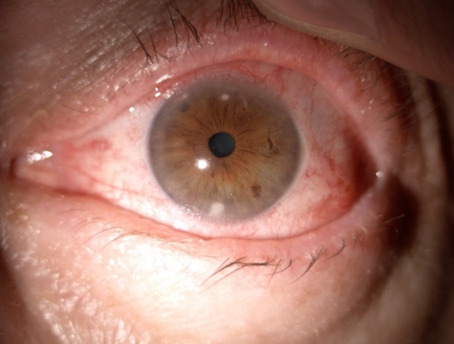
18 days after the first examination, peripheral sterile corneal infiltrates

On the 18th day of follow-up, the BVCA of OD remained 1, while the BVCA of OS was 0,8 with difficulty. IOP was within normal limits in OU, 14 mmHg NCT. Stromal, small infiltrates were observed at 6, 11, and 12 o’clock positions of the peripheral cornea (**Fig. 1**). The diagnosis of peripheral sterile corneal infiltrates was made. The treatment with fluorometholone ophthalmic solution 2 mg/ml in OS was initiated, one drop four times daily for two weeks. 

**Fig. 2:**
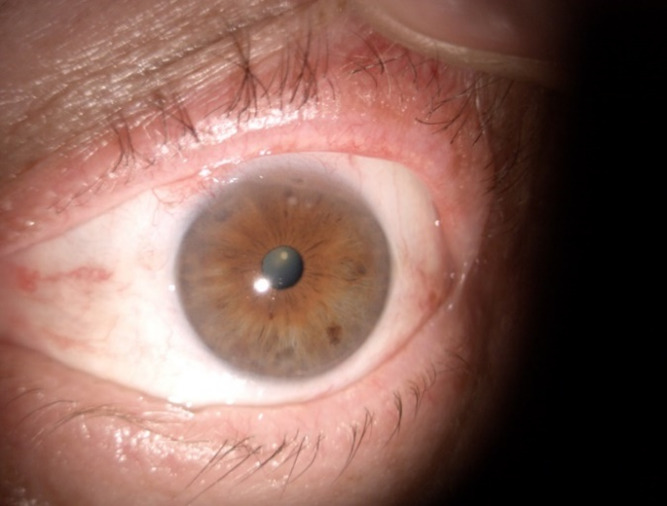
Three weeks after initial presentation

After one week (approximately 3 weeks after the first examination), the BCVA of OU was 1 and IOP was maintained within normal limits. The infiltrates disappeared, leaving only one spot at the 12 o’clock position (**Fig. 2**). The treatment was continued as follows: fluorometholone 2 mg/ml four drops per day for one week, three drops per day for one week, two drops per day for one week and finally one drop per day for one week.

**Fig. 3:**
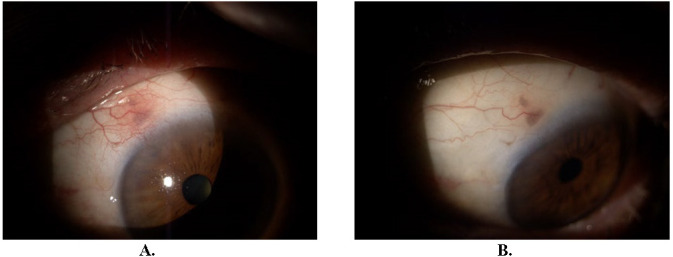
Two months after the initial presentation. Nodular episcleritis (**A**). Epinephrine 10% test (**B**)

Two months after the initial presentation, the patient came for follow-up. BCVA maintained 1 and IOP was slightly elevated in OS, 22 mmHg. At the level of the left eye conjunctiva, at the 11 o’clock position, a hyperemic, mobile nodule was observed (**Fig. 3A**). The epinephrine 10% test was positive (**Fig. 3B**). The diagnosis of nodular episcleritis was made. The treatment with fluorometholone was resumed as follows: 4 drops per day for two weeks then gradually decreased by one drop per week.

**Fig. 4:**
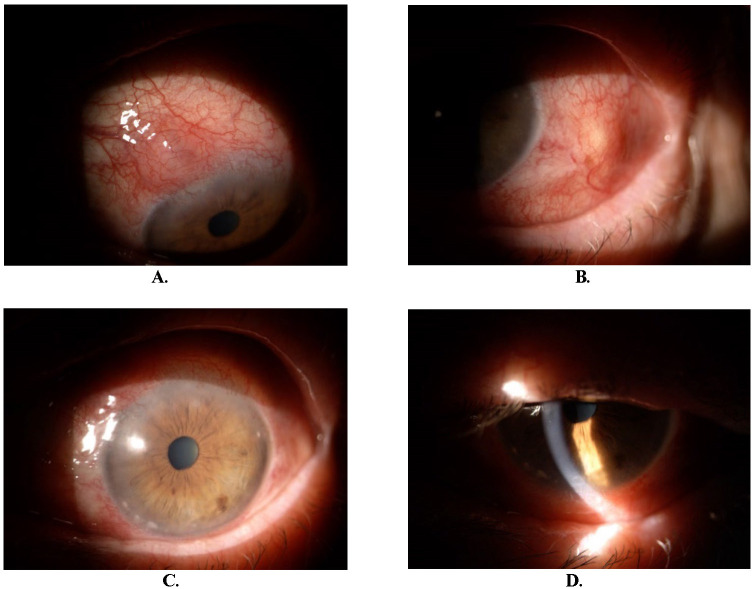
Three months after the initial presentation. Episcleral nodule at 11 (**A**) and 3 (**B**) o’clock positions. Peripheral sterile corneal infiltrates mostly at 7-8 o’clock positions (**C**) and inferior endothelial precipitates (**D**)

 After approximately one month of resumed treatment, the patient presented with ocular discomfort and slightly decreased visual acuity of OS. Ophthalmological examination revealed dilated conjunctival and episcleral vessels, episcleral nodules at 11 and 3 o’clock positions (**Fig. 4A, B**), peripheral sterile corneal infiltrates mostly at 7-8 o’clock positions (**C**), and inferior endothelial precipitates (**D**).

BCVA was 1 for OD and 0,9 for OS, IOP for OD was 15 mmHg adjusted at a pachymetry of 520 µm and for OS it was 24 mmHg adjusted at a pachymetry of 582 µm. The diagnoses made at that moment were episcleritis and hypertensive anterior uveitis. Treatment was initiated with dexamethasone 1 mg/ml ophthalmic solution 4 drops per day for 28 days, dorzolamide 20 mg/ml, and timolol 5 mg/ml combined ophthalmic solution 2 drops per day for 28 days, and acyclovir tablets 800 mg, 5 per day for 10 days. 

**Fig. 5:**
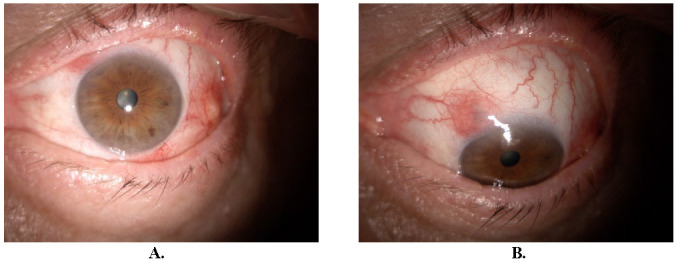
Four months after the initial presentation. Episcleral nodule at 11 and 3 o’clock positions (**A, B**)

The evolution was favorable, endothelial precipitates disappeared after five days. At one month, episcleral nodules were present (**Fig. 5A, B**). BCVA was 1 for OD and maintained 0,9 for OS. IOP was within normal limits. The patient continued treatment with dexamethasone and dorzolamide with timolol.

**Fig. 6:**
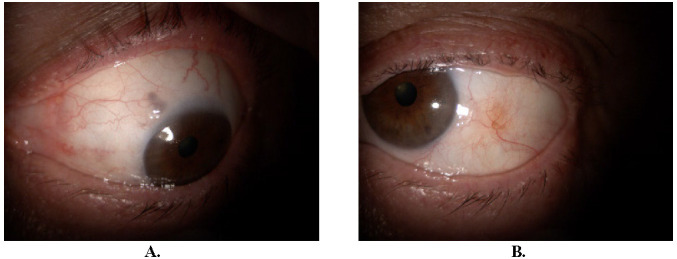
Six months after initial presentation (**A, B**)

After two months, episcleral nodules disappeared (**Fig. 6A, B**). BCVA was 1 for both eyes. 

## Discussion

*Herpes zoster ophthalmicus*

Herpes zoster ophthalmicus occurs when the reactivation of the dormant virus involves the ophthalmic division (V1) of the trigeminal nerve (V). The manifestations can only include the specific periocular rash and pain, but some patients present with direct ocular involvement [**5**]. 

*Ocular manifestations*

The spectrum of symptoms and signs affecting the eye may vary during different stages of the disease. Some of the most common symptoms are eye pain, red eye, tearing, blurry vision, and foreign body sensation [**5**]. The most frequent ocular presentations are conjunctivitis, keratitis, uveitis, episcleritis, and scleritis [**2**,**6**]. Corneal involvement includes epithelial, stromal, and endothelial keratitis. Uveitis can be anterior, posterior or panuveitis [**7**]. Some uncommon manifestations may be acute retinal necrosis, external ocular motor palsies, and optic neuritis [**8**].

*Treatment*

It consists of antiviral therapy, to limit the replication of the virus. Acyclovir 800 mg is administrated five times per day orally, which should be initiated within 72 hours of the clinical presentation for at least 7 days [**8**]. Alternatives are valacyclovir 1000 mg and famciclovir 500 mg, both administrated three times per day orally for at least 7 days. The last two require renal dosing. Immunocompromised adults require intravenous administration of acyclovir, 10 mg/kg of ideal body weight, every eight hours for at least 7 days, or foscarnet 90 mg/kg every 12 hours [**7**]. The following treatment options for specific ocular manifestations are recommended: conjunctivitis requires topical lubrication and antibiotics for secondary bacterial infection prevention; epithelial keratitis requires topical lubrication; stromal keratitis requires topical steroids; episcleritis and scleritis require topical nonsteroidal anti-inflammatory agents and/or steroids; uveitis requires topical and oral steroids, oral antivirals (e.g. acyclovir) and cycloplegics; acute retinal necrosis and progressive outer retinal necrosis require intravenous antivirals (e.g. acyclovir) followed by oral antivirals (e.g. acyclovir) [**5**,**9**].

*Prognosis*

The variability is high, and it is based on the patient’s risk factors, treatment initiation time, and severity of the disease [**7**]. 

*Complications*

In a study of 869 patients, the complications were: corneal scar, neurotrophic keratopathy, band keratopathy, corneal melt, corneal perforation, iris transillumination defects, acute retinal necrosis, high intraocular pressure, glaucoma, moderate and severe vision loss. On multivariate analysis, factors associated with moderate visual loss (≤ 20/50) were: older age, white ethnicity, presenting visual acuity, and uveitis. On multivariate analysis, factors associated with severe visual loss (≤ 20/200) were: older age, immunosuppression, presenting visual acuity, and uveitis [**10**]. A frequent neurological complication is a postherpetic neuralgia. It is characterized by powerful pain that persists for three or more months after the onset of the disease [**2**,**11**]. The most vulnerable patients are the ones older than or equal to 50 years of age, resulting in an important correlation between increasing age and the occurrence of postherpetic neuralgia [**11**].

*Vaccination*

Prevention of the diseases is effective with the help of vaccines. Nowadays, two vaccines are available. The first one is the live attenuated Zostavax vaccine (ZVX), which is given as a subcutaneous injection and is a one-time vaccination. The second one is a non-live recombinant vaccine, named Shingrix, which is given as two intramuscular injections, 2 to 6 months apart [**1**,**12**]. The overall efficacy against the incidence of herpes zoster is 51.3% for ZVX and 97.2% for Shingrix. Both are recommended for immunocompetent adults aged ≥ 50 years. Shingrix is also recommended for adults aged ≥ 18 years, who are at increased risk of herpes zoster due to immunodeficiency or immunosuppression [**13**].

## Conclusion

A 70-year-old patient diagnosed with herpes zoster ophthalmicus maintained a visual acuity of 1 despite numerous late ophthalmic manifestations. 

**Conflict of Interest Statement**

The authors state no conflict of interest. 

**Informed Consent and Human and Animal Rights Statement**

Informed consent has been obtained from the patient included in the case report. 

**Authorization for the use of human subjects**

Not applicable. 

**Acknowledgments**

None. 

**Sources of Funding**

None. 

**Disclosures**

None. 
